# HIF-1α Inhibits Wnt Signaling Pathway by Activating Sost Expression in Osteoblasts

**DOI:** 10.1371/journal.pone.0065940

**Published:** 2013-06-11

**Authors:** Dafu Chen, Yang Li, Zhiyu Zhou, Chengai Wu, Yonggang Xing, Xuenong Zou, Wei Tian, Chi Zhang

**Affiliations:** 1 Laboratory of Bone Tissue Engineering, Beijing Research Institute of Traumatology and Orthopaedics, Beijing JiShuiTan Hospital, Beijing, China; 2 Bone Research Laboratory, Texas Scottish Rite Hospital for Children, University of Texas Southwestern Medical Center, Dallas, Texas, United States of America; 3 Department of Spine, The First Affiliated Hospital of Sun Yat-sen University, Guangzhou,China; Georgia Regents University, United States of America

## Abstract

The nature of the cellular and molecular mechanisms for the transition of avascular cartilage replacement with bone during endochondral ossification remains poorly understood. One of the driving forces is hypoxia. As a master regulator of hypoxia, hypoxia-inducible factor-1α (HIF-1α) has been reported to couple angiogenesis to osteogenesis. Our recent study has demonstrated that osteoblast growth is inhibited under hypoxia and that HIF-1α cooperates with Osterix (Osx) to inhibit Wnt pathway. However, molecular mechanisms for inhibitory effects of HIF-1α on Wnt pathway are not well understood. In this study, our quantitative RT-PCR results revealed that the expression of a Wnt antagonist Sclerostin (Sost) was upregulated in osteoblasts during hypoxia while HIF-1α was upregulated. Treatment of desferrioxamine (DFO), a HIF-1α activator, led to further increase of Sost expression, suggesting that HIF-1α may activate Sost expression. The regulation of Sost gene expression by HIF-1α was then investigated. We performed loss-of-function experiments to examine Sost expression by using siRNA approach against HIF-1α, and found that the inhibition of HIF-1α by siRNA in osteoblasts led to the decrease of Sost expression. To address transcriptional regulation of Sost gene by HIF-1α, transient transfection assay was performed and showed that HIF-1α activated *Sost-1 kb* promoter reporter activity in a dose-dependent manner. To narrow down the minimal region of *Sost* promoter activated by HIF-1α, we generated a series of deletion mutants of *Sost* constructs. It was demonstrated that Sost-260 was the minimal region of *Sost* promoter for HIF-1α activation and that Sost-106 construct, which lack hypoxia response element, abolished HIF-1α-mediated Sost reporter activation. Gel shift assay showed that HIF-1 bound to the promoter sequence of Sost directly. These findings support our hypothesis that HIF-1α activates Sost expression. This study provides a novel molecular mechanism through which HIF-1α inhibits Wnt signaling in osteoblasts.

## Introduction

Bone formation includes two distinct processes: endochondral ossification which requires a cartilage intermediate and intramembranous ossification which forms directly from mesenchymal condensations without cartilage template. Bone formation is a highly regulated developmental process involving the osteoblast differentiation from mesenchymal stem cells. Osteoblast differentiation is controlled by different important transcription factors and signaling proteins, including Indian Hedgehog, Runx2, Osterix (Osx), and Wnt pathway [1], [2]. The observation that Osx inhibits the Wnt pathway highlights the potential for novel feedback control mechanisms involved in bone formation [3]. Replacing the avascular cartilage template with highly vascularized bone is the key step of endochondral ossification. During endochondral bone formation, chondrocytes model the growth plate at the long bone distal ends and become hypertrophic and hypoxic. Growth plate chondrocytes go through well-ordered and regulated phases of cell proliferation, differentiation, and apoptosis [4], [5]. Differentiation is followed by hypertrophic chondrocyte death, blood vessel invasion, and replacement of the cartilage matrix with a trabecular bone matrix. Angiogenesis and osteogenesis are coupled spatially and temporally in bone formation [6]. The nature of the cellular and molecular mechanisms for the transition of avascular cartilage replacement with bone remains poorly understood. One of the driving forces is hypoxia. Hypoxia-inducible factor-1α (HIF-1α) is a master regulator of cellular response to hypoxia. For endochondral ossification, HIF-1α activates VEGF, and causes enhanced bone modeling [7]. It has been speculated that the hypoxia in the chondrocytes imposes energetic limitations on the cells as they evolve from a proliferative to a terminally differentiated state [8].

Wnt signaling has been studied for its broad range of activities in cell proliferation, differentiation and cell death during both embryonic development and the adult stage in a variety of tissue types including bone [9]. As secreted glycoproteins, Wnts bind to Frizzled family receptors and low-density lipoprotein receptor-related proteins (LRP) 5/6 coreceptors. Without Wnt ligands, β-catenin forms a complex with the APC, Axin and the kinases glycogen synthase kinase 3 (GSK3), which facilitates phosphorylation and proteosomal degradation of β-catenin. Stimulation of these receptors by Wnts leads to the intracellular molecule β-catenin to accumulate and translocate into the nucleus, where it associates with TCF/Lef1 transcription factor to activate transcription of target genes. It has been known that Wnt/β-catenin pathway play a crucial role in bone formation and bone metabolism [10]. Conditional inactivation of β-catenin in either skeletal progenitor cells or at a later stage of osteoblast development in mouse embryos blocks osteoblast differentiation [11], [12], [13], [14]. The Wnt signaling is also required for normal osteoblast proliferation. When β-catenin is stabilized in osteoblasts during mouse embryonic development a marked increase in osteoblast proliferation occurs [14]. Moreover *Lrp5-*null mice, which phenocopy the osteoporosis-pseudoglioma syndrome in humans [15], develop a phenotype with low bone mass because of decreased osteoblast proliferation [16]. In addition, the Wnt signaling antagonist Dkk1 prevents the activation of Wnt signaling by binding to LRP5/6. The bone formation and bone mass of heterozygous *Dkk1* mutant mice increase with an increased number of osteoblasts [17]. In contrast, the overexpression of *Dkk1* in osteoblasts leads to severe osteopenia with decreased osteoblast numbers [18]. Sclerostin (Sost), another extracellular Wnt antagonist, binds to LRP5/6 receptor to prevent Wnt binding to LRP5/6 and inhibits Wnt signaling [9], [19], [20], [21]. It has been demonstrated that the Sost loss-of-function mutations are the cause for Sclerosteosis and Van Buchem disease with dramatically increased bone mass due to increased Wnt signal [9]. On the other hand, transgenic mice that overexpress *Sost* are osteopenic due to reduced bone formation, consistent with a model whereby *Sost* negatively regulates osteoblast activity [22]. Thus, Wnt/β-catenin signaling stimulates osteoblast proliferation.

Our recent study explored the role of hypoxia/HIF-1α in osteoblast proliferation. We have found that osteoblast growth is inhibited under hypoxia and that HIF-1α inhibits Wnt pathway. Interestingly, Osx and HIF-1α cooperatively inhibit Wnt pathway [23]. However, molecular mechanisms for inhibitory effects of HIF-1α on Wnt pathway are not well understood. In this study, we provided evidences to show that HIF-1α activates Sost expression. This reveals a new molecular mechanism through which HIF-1α inhibits Wnt signaling in osteoblasts.

## Methods

### Plasmid Constructs and Subcloning

PIP2N-HIF-1α plasmid was used as previously described [24]. Jab1 plasmid was used as described [25]. The fragments of Sost promoter region were generated by PCR using mouse genomic DNA as a template and subcloned into the XhoI and MluI sites of pGL-3 vector as previously described [26]. Primers were obtained from Integrated DNA Technologies (IDT) (Coralville, IA), and the sequences were as follows: 1) SOST-Xho-3 5′GCG CCT CGA GTG TCC AGC CTA GAT ACG GTT G, 2) SOST-Mlu-1K-5 5′ GCG CAC GCG TGA AAG ACA CCT CCT CAG GTC 3) Sost-Mlu-540 5′GCG CAC GCG TAA GGC ATC CTT CTG 4) Sost-Mlu-260 5′GCG CAC GCG TTG TGT CCC TGC CTC 5)Sost-Mlu-106 5′GCG CAC GCG TTG AGG AGG AGG GTG A. All constructs including mutants were verified by DNA sequencing.

### Cell Cultures and Hypoxia Experiment

MC3T3 osteoblastic cells (ATCC) were cultured in Alpha Minimum Essential Medium with ribonucleosides, deoxyribonucleosides, 2 mM L-glutamine and 1 mM sodium pyruvate (GIBCO) and supplemented with 10% FBS and penicillin plus streptomycin. HEK293 cells (ATCC) were grown in Dulbecco’s Modified Eagle Medium (GIBCO) supplemented with 10% FBS and 100 units/ml penicillin and 100 ug/ml streptomycin. Cells were cultured in 95% air/5% CO_2_ humidified incubator. Cells were trypsinized and plated before transfection. In hypoxia experiments, MC3T3 cells were maintained in Alpha Minimum Essential Medium, and cultured in normoxic (20%O_2_) or hypoxia (1%O_2_) condition incubator with 5%CO_2_ and the balanced N_2_ before harvest. All endpoints measured in hypoxia cells were compared with those in cells kept under normoxic condition. Desferrioxamine (DFO) was purchased from Sigma (D9533-1G).

### RNA Isolation and Real-time RT-PCR

Total RNA was isolated from MC3T3 osteoblasts with TRIzol reagent (Invitrogen) followed by RNeasy mini kit (Qiagen) as previously described [27]. TaqMan One-Step RT-PCR Master Mix reagent (Applied Biosystems) was used for quantitative RT-PCR. Reaction volume is 50 ul per well on 96-well plates. Analysis was performed with ABI PRISM 7500 sequence detection system (Applied Biosystems). Primers were ordered from Applied Biosystems. Transcript levels were normalized to heat shock protein 90 (HSP90) levels. All reactions were done in duplicate and all experiments were repeated at least three times. The relative mRNA expression levels were calculated according to the comparative C_T_ (ΔΔC_T_) method as described by the manufacturer (User Bulletin #2, Applied Biosystems). Target quantity is normalized to endogenous control and relative to a calibrator, and is calculated using formula: Target amount = 2^−ΔΔC^_T_.

### Protein Purification and Western Blot

Protein was isolated by acetone precipitation from the cell lysates as previously described [28]. The protein pellet was dissolved in 1% SDS buffer, warmed for 15 min at 55°C, and centrifuged for 5 min at 14000 rpm. Protein concentrations in the supernatant were determined using a BCA Protein Assay Kit (Pierce). Proteins were separated on 10% SDS-PAGE gels and transferred to a PVDF membrane followed by Western blot analysis. Briefly, 3% milk in TBS containing 0.1% Tween-20 was used to block non-specific binding. The blot was subsequently incubated with an anti-HIF-1α rabbit polyclonal antibody (1∶200, Abcam), an anti-Sost rabbit polyclonal antibody (1∶60, Abcam), or an anti-HSP90 rabbit polyclonal antibody (1∶200, Abcam) followed by a secondary antibody (peroxidase-conjugated anti-rabbit IgG 1∶5000, Sigma). After each antibody incubation, blots were extensively washed in TBS containing 0.1% Tween-20. For detection, the ECL kit (Amersham Life Sciences) was used according to the directions of the manufacturer.

### siRNA Interference

MC3T3 cells were transfected by siRNA against mouse HIF-1α with Lipofectamine 2000 as previously described [29]. siRNA oligos were purchased from Thermo Scientific Dharmacon, and siGENOME Lamin A/C Control siRNA was used as a non-specific control. Cells were cultured in 6-well plates. One day before transfection, cells were plated in 1 ml of growth medium without antibiotics. Cells were 30–50% confluent at the time of transfection. For each sample, siRNA:Lipofectamine^.^ 2000 transfection complex was prepared as follows: (1) Dilute 2 µl of 50 µM siRNA in 50 µl of Opti-MEM I Reduced Serum Medium without serum; (2) Mix Lipofectamine^.^ 2000 gently, then dilute 3 µl in 50 µl of Opti-MEM I Medium; (3) Combine the diluted siRNA with the diluted Lipofectamine^.^ 2000; (4) Add 100 µl of siRNA:Lipofectamine^.^ 2000 complex to each well. After 4 hours incubation, the growth medium was replaced. Cells were cultured at 37°C in a CO_2_ incubator for 24 hours before harvest.

### Transient Transfection Assay

HEK293 cells were plated in 12-well plates, cultured to 60%–80% confluence and transfected with FuGENE 6 (Roche) as previously described [30]. Cells were cotransfected with 300 ng of Sost promoter reporter, HIF-1α- expression plasmid as indicated and 25 ng of pSV2-beta-gal. After transfection, cells were incubated for 24 h before harvest. The reporter assays were analyzed with BD Monolight system (BD Biosciences). Luciferase activity was normalized by β-galactosidase activity. Every transfection experiment was done at least three times. Values were presented as the mean ±S.D.

### Gel Shift Assay

Gel shift assay and nuclear extracts as the HIF-1 protein resource were prepared as previously described with some modifications [25], [31]. The DNA sequences of the oligonucleotides used for Gel shift assay were as follows: Sost 5′ CAC CCC ACC CCC GTG AGG AGG AGG GTG AGG AAA C. DNA oligonucleotide was labeled using a Biotin 3′ end DNA Labeling Kit (Cat#: 89818, Pierce Biotechnology Inc.). Nuclear extracts were isolated from MC3T3 cells under either normoxia or hypoxia conditions for 16 h. Four µg of nuclear extracts and biotin-labeled DNA probe were incubated in 1x binding buffer for 20 min at room temperature using LightShift Chemiluminescent EMSA kit (Cat#: 20148). Protein–DNA complexes were separated on 4% polyacrylamide gels in 0.5x TBE buffer, and transferred onto Biodyne B Nylon Membrane (Cat#: 77016). The membrane was blocked in 1x blocking buffer, washed five times with 1x wash buffer, and visualized by a Chemiluminescent Nucleic Acid detection Module (Cat#: 89880). Two hundred-fold molar excess of unlabeled Sost promoter oligos was used as specific competitor DNA.

### Statistical Analysis

All experiments were repeated a minimum of 3 times. Data was reported as the mean ± standard deviation (S.D.). Comparisons were made between groups by Student’s t test with p<0.05 being considered as statistically significant.

## Results

### Hypoxia Leads to Upregulation of Sost Gene Expression

**Figure 1:**
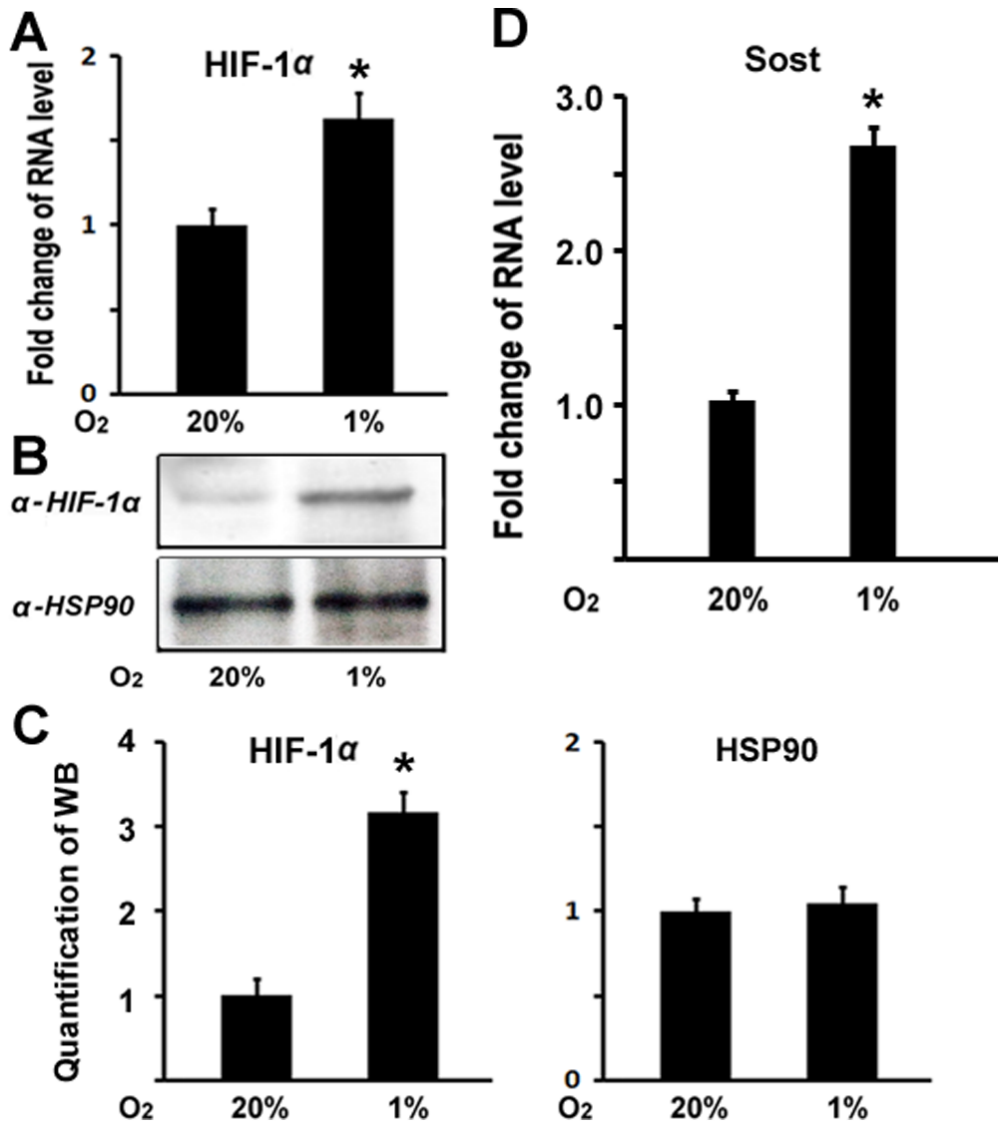
Hypoxia leads to upregulation of Sost gene expression. (A) Increase of HIF-1α expression in RNA level in osteoblasts under hypoxia. RNA level was normalized to heat shock protein 90 (HSP90). A paired *t*-test was performed comparing control group (20% O_2_) and hypoxia group (1% O_2_). *: A star indicates statistical significance compared to control group. (B) Western blotting analysis of HIF-1α expression in protein level in osteoblasts under hypoxia. Heat shock protein 90 (HSP90) was used as a loading control. (C) Quantification of western blotting of HIF-1α and HSP90 expressions in protein levels. Protein level from normoxic condition (20%O_2_) group was normalized to a value of 1. (D) RNA expression level of Sost as determined by quantitative real-time RT-PCR. MC3T3 osteoblasts were cultured for 48 hr under hypoxia (1%O_2_). RNA was isolated and quantitated by real-time RT-PCR. The RNA level from normoxic condition (20%O_2_) group was normalized to a value of 1. Values were presented as the mean ±S.D. A paired *t*-test was performed comparing control group (20% O_2_) and hypoxia group (1% O_2_). *: A star indicates statistical significance compared to control group.

**Figure 2:**
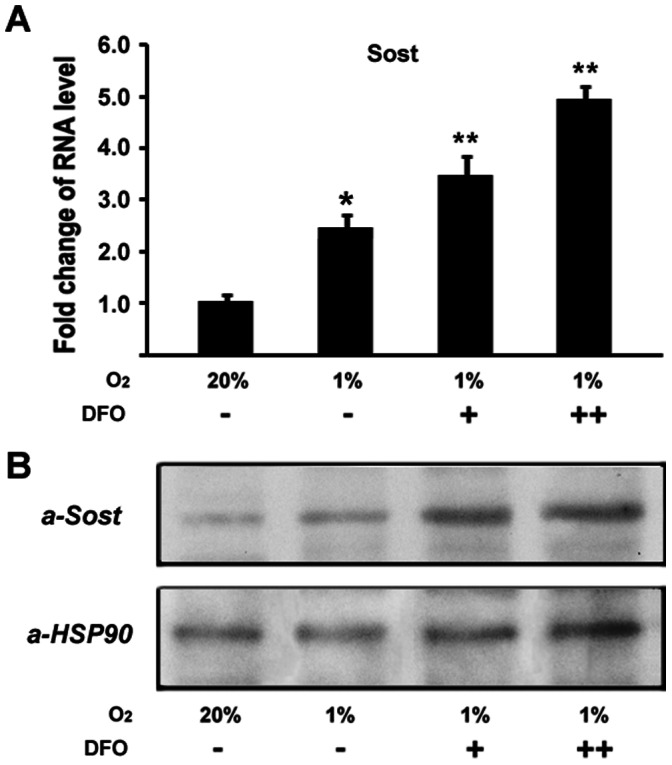
Effect of desferrioxamine on hypoxia-induced Sost expression. (A) RNA expression level of Sost with DFO as determined by quantitative real-time RT-PCR. MC3T3 osteoblasts were cultured for 48 hr under hypoxia (1%O_2_), and treated with desferrioxamine (DFO). +:100 uM; ++:200 uM. The RNA level from normoxic condition (20%O_2_) group was normalized to a value of 1. Values were presented as the mean ±S.D. A paired *t*-test was performed comparing control group (20% O_2_) and hypoxia group (1% O_2_). *: A star indicates statistical significance compared to control group. A paired *t*-test was also performed comparing 1% O_2_ group and DFO group (+ and ++). **: Two stars indicate statistical significance compared to 1% O_2_ group. (B) Western blotting analysis of Sost expression in protein level in osteoblasts under hypoxia. Heat shock protein 90 (HSP90) was used as a loading control.

Our recent studies have demonstrated that hypoxia/HIF-1α inhibits Wnt pathway in osteoblasts, a possible mechanism for hypoxia to inhibit osteoblast proliferation [23]. However, the mechanisms of hypoxia/HIF-1α inhibition on Wnt signaling are not well understood. To explore the possible mechanisms, we used quantitative real-time RT-PCR to examine the changes of gene expressions under hypoxia. MC3T3 osteoblastic cells were cultured and maintained in normoxic (20%O_2_) or hypoxia (1%O_2_) condition under a humidified hypoxia incubator. Total RNA was purified 48 hr following culture in the presence or absence of hypoxia. As shown in Figure 1A, HIF-1α RNA expression was enhanced by 1.6 fold under hypoxia compared with normoxia, and western blotting experiments indicated that HIF-1α protein expression level also increased under hypoxia (Figure 1B). These confirm the hypoxia-mediated upregulation of HIF-1α expression. As a negative control, western blotting experiments indicated that HSP90 protein expression level remained unchanged under hypoxia (Figure 1B). Western blotting results of HIF-1α and HSP90 were quantified in Figure 1C. Interestingly, Wnt signaling antagonist *Sost* expression was found upregulated by about 2.6 folds in osteoblasts under hypoxia, compared with normoxia (Figure 1D). The upregulation in *Sost* RNA level under hypoxia suggests that hypoxia activates *Sost* gene expression. We then asked if the effect of hypoxia on *Sost* expression is related to HIF-1α. To address this question, we used desferrioxamine (DFO) in this assay, a potent HIF-1α activator. Sost expression was then examined under hypoxia. As shown in Figure2A, additions of DFO further increased *Sost* expression under hypoxia in a dose-dependent manner. Western blotting experiments confirmed the Sost expression upregulation by DFO in the protein levels as shown in Figure 2B. These data suggest that HIF-1α is involved in hypoxia-mediated activation of Sost expression.

### Inhibition of HIF-1α by siRNA Leads to Repression of Sost Expression

**Figure 3:**
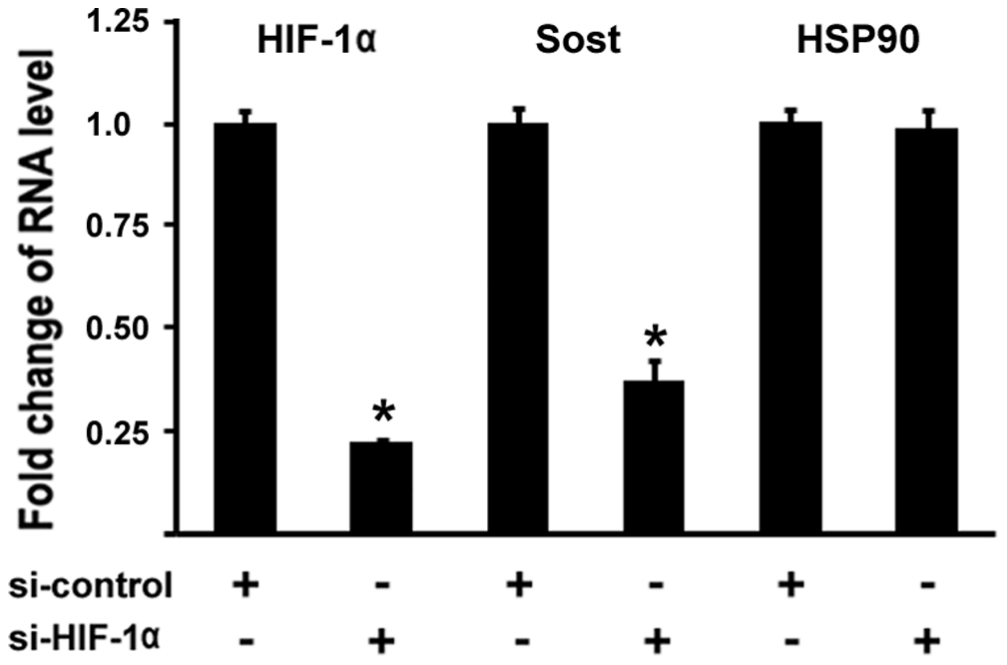
Inhibition of HIF-1α by siRNA results in downregulation of Sost expression in osteoblasts. MC3T3 osteoblasts were transfected with siRNA control or siRNA against HIF-1α. RNA was isolated 24 hr post-transfection and quantitated by quantitative real-time RT-PCR for HIF-1α and Sost, and HSP90 was used as a negative control. The RNA level from the control siRNA group was normalized to a value of 1. Values were presented as the mean ±S.D. si-control: si-RNA control; si-HIF-1α: si-RNA against HIF-1α. A paired *t*-test was performed comparing si-control group and si-HIF-1α group. *: A star indicates statistical significance compared to control group.

To further examine the effect of HIF-1α on *Sost* expression, we used siRNA to knockdown HIF-1α expression in MC3T3 cells. MC3T3 osteoblastic cells were transfected by siRNA against HIF-1α and cultured in hypoxia (1%O_2_) condition under a humidified hypoxia incubator. Real-time RT-PCR was performed to analyze gene expression levels. As shown in Figure 3, when HIF-1α RNA expression was decreased by 79% using siRNA targeted against HIF-1α, *Sost* RNA levels were reduced by approximately 67%. On the other hand, the expression of an unrelated gene HSP90 remained unchanged. We used Lamin A/C Control siRNA as a non-specific control to show the specificity in Figure 3. Therefore, these loss-of-function experiments support a role for HIF-1α in regulating *Sost* gene expression positively.

### HIF-1α Activates the Sost Promoter Activity in a Dose-dependent Manner

**Figure 4:**
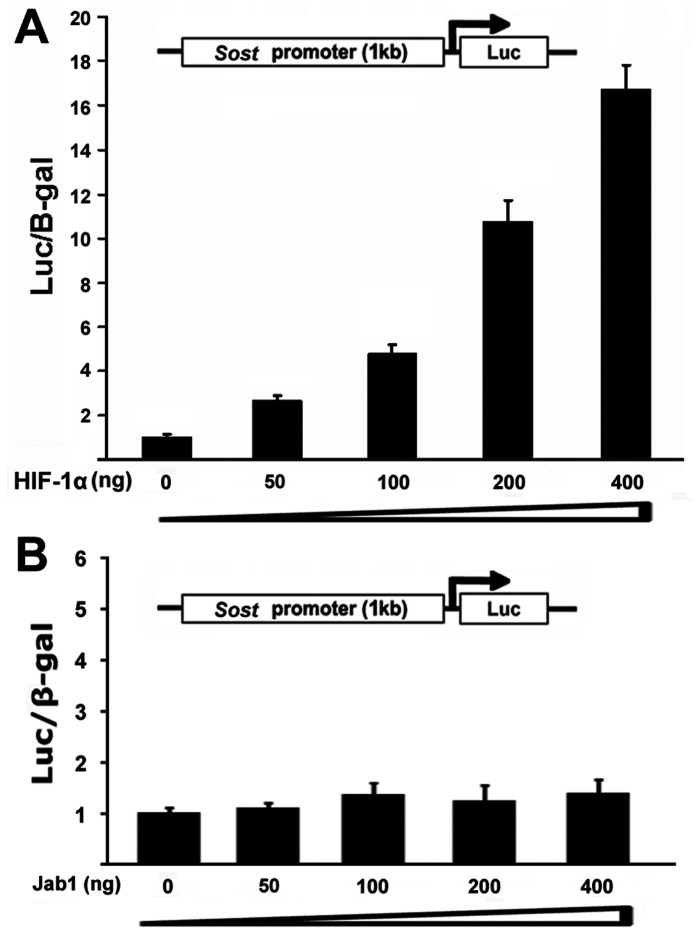
Effect of HIF-1α on Sost promoter activity. (A) HIF-1α activates the *Sost* promoter in a dose-dependent manner. HEK293 cells were transfected with a 1 kb *Sost* promoter-luciferase reporter gene without or with increasing amounts of an HIF-1α-expression plasmid as indicated. Luciferase activity was normalized by β-galactosidase activity. Values are presented as the mean ±S.D. (B) Jab1 does not activate *Sost* promoter activity. HEK293 cells were transfected with a 1 kb *Sost* promoter-luciferase reporter gene without or with increasing amounts of a Jab1-expression plasmid as indicated. Luciferase activity was normalized by β-galactosidase activity. Values are presented as the mean ±S.D.

To determine the direct effect of HIF-1α on the *Sost* promoter activity, we did subcloning to generate a luciferase reporter construct driven by 1 kb *Sost* native promoter. HEK293 cells were transiently transfected with the *Sost* promoter-luciferase reporter and HIF-1α expression vector. HEK293 cells were chosen because they are very easy to grow and have high transfection efficiency for transient transfection assay used in our current study. As shown in Figure 4A, the activation of *Sost* promoter reporter by HIF-1α was detected when as low as 50 ng of HIF-1α was transfected. Increasing amounts of HIF-1α induced markedly higher *Sost* promoter activities, and the transfection with 400 ng HIF-1α resulted in a 16.6-fold increase of *Sost* promoter activity. We used non-specific expression vector Jab1 as a control. There was no effect of Jab1 on *Sost* reporter expression as shown in Figure 4B. These observations demonstrated that HIF-1α stimulated 1 kb *Sost* promoter luciferase reporter in a dose-dependent manner, suggesting that HIF-1α transcriptionally activated the expression of *Sost* gene.

### Identification of the Minimal Region in the Promoter of Sost Gene for HIF-1α Activation

**Figure 5:**
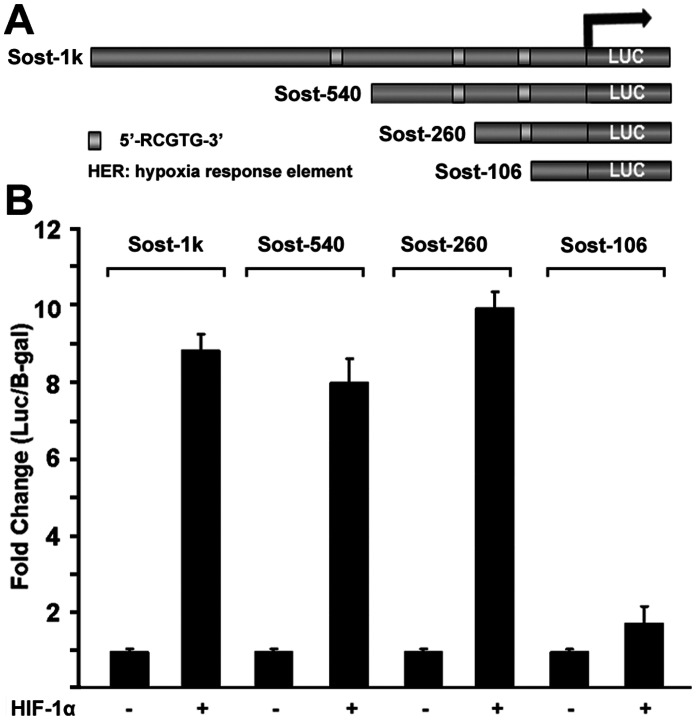
Identification of the minimal region in the promoter of Sost gene for HIF-1α activation. (A) Schematic representation of the *Sost* deletion mutants. HRE: hypoxia response element. Sost-1 kb, Sost-540 bp, Sost-260 bp and Sost-106 bp *Sost* promoter reporter plasmids were constructed, using luciferase (LUC) as a reporter. (B) Deletion analysis of the *Sost* promoter-reporter constructs. Sost-1 kb, Sost-540 bp, Sost-260 bp and Sost-106 bp promoter-reporter plasmids (300 ng each) were cotransfected with 200 ng of the HIF-1α expression plasmid in HEK293 cells. Twenty-four hours post-transfection, cell extracts were prepared and analyzed for luciferase activity. Luciferase activity was normalized by β-galactosidase activity. Values are presented as the mean ±S.D.

Our data have shown HIF-1α can stimulate *Sost* promoter activity, however it is still not clear which region within *Sost* promoter is responsible for HIF-1α activation. To address this, we first searched the hypoxia response element (HRE) in the *Sost* promoter. It is known that the HRE core sequence is 5′-RCGTG-3′, which is critical for HIF-1 binding. According to the sequence analysis of *Sost* promoter, there are three potential HRE within 1 kb *Sost* promoter region. To determine the minimal region of *Sost* promoter which can be regulated by HIF-1α, *Sost* luciferase reporter constructs driven by different lengths of *Sost* promoter region were generated as shown in Figure 5A. Sost-1 kb, Sost-540 bp and Sost-260 bp constructs all contain HRE. Transient transfection assay were carried out to narrow down responsible region within 1 kb *Sost* promoter for HIF-1α activation. As shown in Figure 5B, 200 ng of HIF-1α was able to activate *Sost* promoter reporter expression of Sost-1 kb, Sost-540 bp and Sost-260 bp in transient transfection assay by 8.8 fold, 7.9 fold and 9.9 fold, respectively. However, HIF-1α activation was almost abolished in Sost-106 bp reporter, which lack HRE. These data suggest that the HRE in the promoter region between Sost-260 bp and Sost-106 bp is responsible for critical binding for HIF-1α. Thus, these results indicated that Sost-260 bp is the minimal region of Sost promoter in this study which is responsible for *Sost* promoter activation by HIF-1α.

### Binding of the HIF-1 Complex to the HRE Site in the Proximal 260 bp Region of Sost Promoter

**Figure 6:**
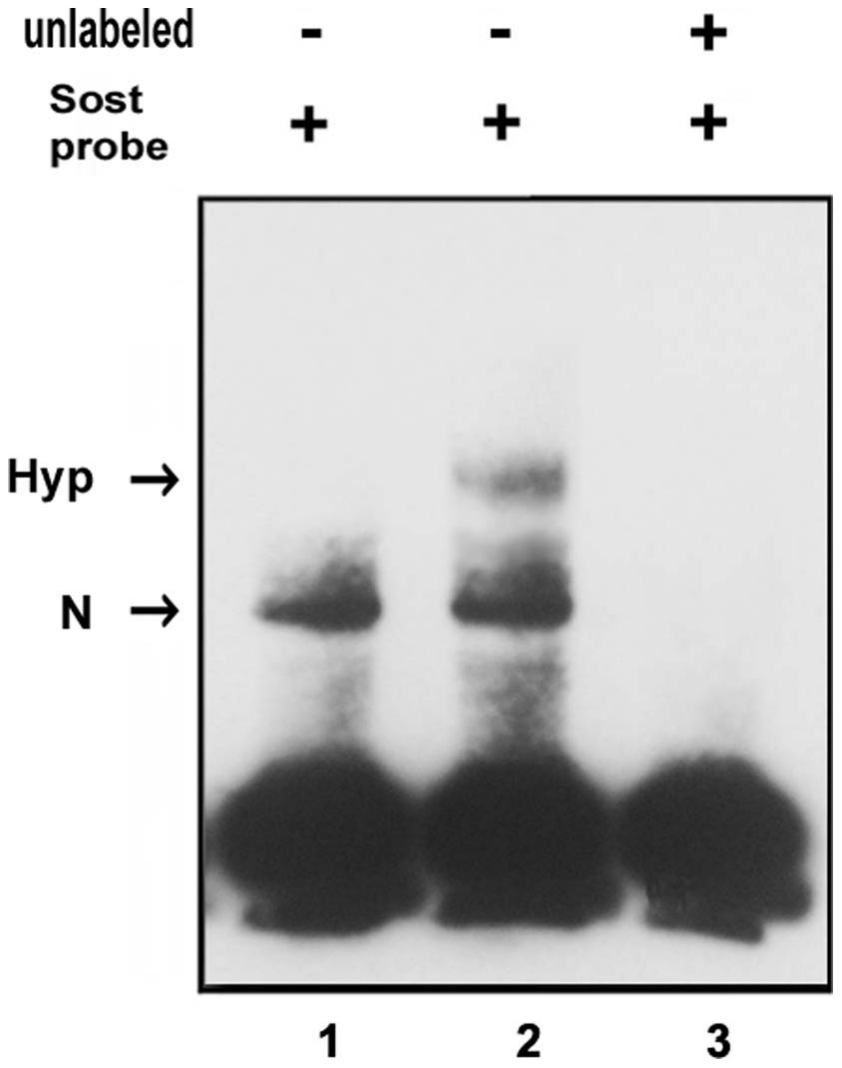
Binding of the HIF-1 complex to the HRE of Sost promoter under hypoxia. Nuclear extracts were isolated from MC3T3 cells in either normoxia or hypoxia conditions from 16 h and used as the HIF-1 protein resource. DNA oligonucleotides of *Sost* were labeled by Biotin. Nuclear extracts and biotin-labeled DNA probe were incubated. Protein-DNA complexes were separated on 4% polyacrylamide gels, and visualized by a Chemiluminescent Nucleic Acid detection Module. Complexes observed in extracts under normoxia (N, lane 1) or hypoxia conditions (Hyp, lane 2) are indicated by the arrows. Two hundred-fold molar excess of unlabeled *Sost* promoter oligos were used under hypoxia condition (lane 3).

To determine whether the HRE site in the proximal minimal region of *Sost* promoter is capable of binding with HIF-1 complex, Gel shift assay was performed. MC3T3 cells cultured in normoxia or hypoxia were harvested, lysed, and nuclear extracts were prepared. Nuclear extracts under hypoxia has successfully been used as the HIF-1 protein resource in Gel shift assay [31]. As shown in Figure 6, hypoxia resulted in the appearance of a distinct complex with a retarded electrophoretic migration that appears as a single band (lane 2). This HIF-1 complex was abolished by excess unlabeled *Sost* promoter oligos (lane 3), which was used to test the binding specificity. The data indicated that hypoxia-induced HIF-1 complex bound to *Sost* promoter oligos specifically.

## Discussion

Our recent observations have indicated that hypoxia/HIF-1α inhibit osteoblast proliferation, and that HIF-1α has a synergistic effect with Osx on the inhibition of Wnt pathway [23]. Because of the role of Wnt pathway in stimulating osteoblast proliferation, we speculate that hypoxia-induced inhibition of osteoblast proliferation may be at least partially through inhibition of Wnt pathway by HIF-1α. However, the mechanisms of hypoxia/HIF-1α inhibition on Wnt signaling are not well known. In this study, we performed a series of experiments to study the effect of HIF-1α on the extracellular Wnt antagonist Sost. We provide evidences to demonstrate that HIF-1α activates Sost expression, a novel mechanism of HIF-1α inhibitory effect on Wnt signaling pathway in osteoblasts.

Wnt signaling is known to have the major impact at different stages of bone formation and bone metabolism [9], [10]. Wnt signaling-mediated gene expression can promote osteoblast proliferation and differentiation. Some studies investigated the role of Wnt/β-catenin signaling in nonunion and osteoporosis, suggesting Wnt signaling could possibly have potential to become a target of pharmacological intervention to increase bone formation [32], [33]. Sost is one of the Wnt antagonists. The Sost loss-of-function mutations in human cause the autosomal recessive bone dysplasias Sclerosteosis and Van Buchem disease, which are characterized by progressive bone overgrowth throughout life, enlargement of the jaw and facial bones, and increased bone formation [20], [34].

HIF-1α is the crucial mediator of the adaptive response of cells to hypoxia. The oxygen dependent degradation of HIF-1α is controlled by a family of HIF prolyl hydroxylases. Under normoxic conditions, HIF-1α is hydroxylated by prolyl hydroxylases that act as oxygen sensors. Hydroxylation of specific proline residues on HIF-1α is followed by proteasomal degradation. Under hypoxic conditions, HIF-1α is stabilized, translocated to the nucleus, and forms a heterodimer with HIF-1β to regulate target genes. These target genes are involved in a variety of cellular processes including angiogenesis, energy metabolism, cell proliferation and survival, vasomotor control, and matrix metabolism [35]. It has been shown that constitutive activation of the HIF-1α pathway in mice promotes robust bone modeling and acquisition in long bones, and conversely, loss of *HIF-1α* in osteoblasts results in narrow, less vascularized bones [7]. These results suggest that HIF-1α is critical for coupling angiogenesis to osteogenesis during long bone formation. Osteoblasts reside on the nascent bone surface and sense reduced oxygen or nutrient levels, and HIF-1α is an important mediator in this process.

The current study addresses possible mechanisms for hypoxia/HIF-1α to inhibit Wnt pathway. This study indicates that HIF-1α-mediated Sost activation is one of possible mechanisms for hypoxia/HIF-1α to inhibit Wnt pathway. This is supported by several evidences: 1) quantitative RT-PCR results showed that *Sost* gene was upregulated along with HIF-1α under hypoxia; 2) the treatment of HIF-1α activator DFO further enhanced the expression of *Sost* gene; 3) the inhibition of HIF-1α by siRNA in osteoblasts led to the expression decrease of *Sost* gene; 4) our transfection assay showed that HIF-1α activated Sost promoter reporter activity. A rescue experiment on cell growth by overexpressing Sost in HIF-1a knockdown cells could help to address the function activity further in the future. However, our study cannot rule out other possible mechanisms of the inhibitory effect of hypoxia/HIF-1α on Wnt signaling pathway.

In summary, HIF-1α activates the expression of Wnt antagonist Sost gene. This provides a novel mechanism through which HIF-1α inhibits Wnt signaling pathway in osteoblasts. Elucidation of HIF-1α inhibition of Wnt signaling will help to better understand the molecular mechanism of HIF-1α effect on osteoblasts.
